# Editorial 2021

**DOI:** 10.4102/sajp.v77i1.1633

**Published:** 2021-12-03

**Authors:** Aimée V. Stewart

**Affiliations:** 1 Department of Physiotherapy, School of Therapeutic Sciences, Faculty of Health Sciences, University of the Witwatersrand, Johannesburg, South Africa

We are now approaching the end of the second year of the coronavirus disease 2019 (COVID-19) pandemic, and we are still living differently from our usual lives. We have had to make adaptations to our working, teaching, research and our interactions with colleagues, friends and families. Everyone has made sacrifices, some more than most, as we learn to navigate the travails of this pandemic. This year has been as difficult as 2020 with two waves of the virus. Having said that, it is important to remember that we can be vaccinated against the virus, and that the more people there are who are vaccinated, the more control of the pandemic there will be.

Despite the difficulties that we are all experiencing, the journal has continued to thrive. For this, we are thankful to our authors who keep the submissions rolling in, our reviewers who do a sterling job in carefully considering the merits and the issues in the submissions, and our publishers who keep the publication process on track.

We are delighted to welcome Prof. Verusia Chetty from the University of KwaZulu-Natal as our new Associate Editor. Prof. Josè Franz is taking a well-earned rest after many years at the helm of our journal.

Again, I am glad to report that we are receiving submissions from an increasingly wide range of authors from South Africa and other countries. In addition, we have accepted several articles from other healthcare professionals who have submitted articles relevant to our profession. I hope that this trend persists as it means that our journal has much broader appeal. We are also publishing a special edition this year namely, ‘Spinal Deformities – Evidence and Patient Safety in Management’ from a group of international researchers who work in the management of spinal deformities.

Prof. Saul Cobbing from the University of KwaZulu-Natal made an important point in his state-of-the-art article published this year (Cobbing 2021): *there is a need for our assessments and interventions to be appropriate and context specific for our many communities that have such stark -socio-economic and cultural differences* (*paraphrased*). These changes to the way in which we manage our patients should be grounded in appropriate studies that determine the effectiveness of interventions that take into consideration the cultural, socio-economic and geographical context of our patients. How can the relatively few physiotherapists in South Africa contribute meaningfully to our total population requiring our expertise? Should we be considering more home programmes, more involvement of the families and caregivers of patients with long-term disabilities, more use of telemedicine and task shifting to name but a few ways of managing patients appropriately in our environment. These are the questions and issues that I hope our researchers and clinicians start considering as we always seek to ensure that we are providing healthcare that is appropriate and relevant to our own population. I hope to see increasing numbers of submissions to our journal that reflect our researchers’ efforts to modify our interventions so that we ensure that our expertise gets to our population that needs it most. This is a worthwhile challenge and one, which I believe our researchers and clinicians need to engage in.

The *South African Journal of Physiotherapy* is going from strength to strength; but to keep improving we need good quality submissions, careful reviewers and continual improvement of the publication systems. The South African Society of Physiotherapy financially sponsors our journal, which is an enormous contribution to its viability. We have a unique opportunity to develop the journal even further as we publish studies that grapple with the given issues and that provide appropriate answers to ensure the relevancy of our profession within our South African healthcare sector.

Our best wishes for 2022 with the hope that the new year does indeed bring some relief from the COVID-19 pandemic.


Prof. Aimée Stewart Editor-in-Chief
*South African Journal of Physiotherapy*

